# Comprehensive Virus Detection Using Next Generation Sequencing in Grapevine Vascular Tissues of Plants Obtained from the Wine Regions of Bohemia and Moravia (Czech Republic)

**DOI:** 10.1371/journal.pone.0167966

**Published:** 2016-12-13

**Authors:** Aleš Eichmeier, Marcela Komínková, Petr Komínek, Miroslav Baránek

**Affiliations:** 1 Mendeleum - Department of Genetics, Faculty of Horticulture, Mendel University in Brno, Lednice, Czech Republic; 2 Crop Research Institute, Ruzyně, Czech Republic; Julius Kühn-Institut, GERMANY

## Abstract

Comprehensive next generation sequencing virus detection was used to detect the whole spectrum of viruses and viroids in selected grapevines from the Czech Republic. The novel NGS approach was based on sequencing libraries of small RNA isolated from grapevine vascular tissues. Eight previously partially-characterized grapevines of diverse varieties were selected and subjected to analysis: Chardonnay, Laurot, Guzal Kara, and rootstock Kober 125AA from the Moravia wine-producing region; plus Müller-Thurgau and Pinot Noir from the Bohemia wine-producing region, both in the Czech Republic. Using next generation sequencing of small RNA, the presence of 8 viruses and 2 viroids were detected in a set of eight grapevines; therefore, confirming the high effectiveness of the technique in plant virology and producing results supporting previous data on multiple infected grapevines in Czech vineyards. Among the pathogens detected, the *Grapevine rupestris vein feathering virus* and *Grapevine yellow speckle viroid 1* were recorded in the Czech Republic for the first time.

## Introduction

The infection of plant tissues by viral pathogens can cause significant economic losses to agriculture crops [1]. Many different viral pathogens occur, especially on vegetatively propagated crops, which have accumulated these pathogens over the centuries and millennia. Pathogen-specific detection techniques, either immunoenzymatic or nucleic acid-based methods, cannot get the overall picture of all of the pathogens present in the plants tested, where each sample must be tested for several pathogens simultaneously. When more pathogens with economical impacts occur in a tested crop in a given region, and needs to be tested for, such a procedure starts to be laborious and inconvenient. This is the case with grapevines, which host about 70 viral pathogens, several viroids, and phytoplasmas. Recently published results have proven that the presence of just a single virus in a single grapevine, or even in a single tissue, is a very rare event [2–5]. The NGS technique (next generation sequencing, or deep sequencing) represents a new level in virological research, especially in diagnostics. This approach now makes possible studying the overall “virome”, the complex of all viruses and viroids present within plant tissues, single vines, or entire vineyards [5].

The availability of such a broad-spectrum detection technique provides powerful support to certification processes, which are supposed to guarantee the clean health status of propagation materials [6].

In addition, the use of the metagenomic approach in vegetatively propagated species would help in preventing the spread of viruses [7] as well as in the identification of quarantine pathogens.

The NGS method generates a very large amount of sequencing data from each sample, and thus the high demand for proper data processing and analysis arises. UNIX-based open-source platforms for the entire process exist, as well as commercial ones such as CLC Genomic Workbench, Geneious, or Lasergene. The need for reference sequences is primarily provided by the NCBI, with its large and freely available databases (https://www.ncbi.nlm.nih.gov/guide/all/). Genomic resources specific to viruses can be accessed at https://www.ncbi.nlm.nih.gov/genome/viruses/.

For plant virologists, deep sequencing is a powerful technology that provides rapid and exhaustive information on the infectious agents (viruses and viroids) present in plant tissues [4, 8]. Therefore, this technology is increasingly being used for the quick identification of viruses replicating in plant tissues, starting either from the analysis of small interfering RNA (sRNA) populations [4], or from sequenced libraries of fragmented double-stranded RNAs (dsRNAs) of viral origins [2, 5], extracted from infected tissues.

Techniques for the molecular detection of grapevine viruses are based on the analysis of RNA, isolated either from vascular tissues or from softer tissues such as leaves and petioles. NGS techniques more frequently use RNA isolated from leaves and petioles as the starting materials [2, 9].

The present work was aimed at demonstrating the NGS technique's utility for the detection of grapevine viruses using phloematic tissues as the starting material, and in obtaining a picture of the virome of different Czech grapevine cultivars.

## Materials and Methods

### Access to samples

All plants analyzed in the work belong to the public collection of the Crop Research Institute, Prague, Czech Republic. They are officially recognized as genetic resources under “The National Program on the conservation and utilization of genetic resources of microorganisms important for food and agriculture”. Their free use for research purpose is guaranteed by the Czech national legislation, namely Act no. 148/2003 Coll., on the conservation and utilization of genetic resources of plants and microorganisms important for food and agriculture.

### Plant samples

Grapevine cuttings were collected in November 2013 from grapevines maintained under controlled conditions in a screenhouse of the Crop Research Institute, Prague, Czech Republic, ensuring freedom from infection of some additional pathogens.

All the grapevines had already been partially characterized, and the present work was focused on those plants known to contain larger numbers of viruses, as well as those showing symptoms of an unclear etiology. Further criteria for plant selection were their having been different cultivars and having different origins from divergent parts of the Czech Republic. A descriptive list of the samples is provided in Table 1.

**Table 1:** A list of tested grapevines including their geographical origin, number of unique sequencing reads, and list of viruses and viroids detected by NGS.

Reference # in the study	Reference # of the plant	Cultivar	Grapevine growing region	Number of unique reads	Viruses detected by NGS	Viroids detected by NGS
G1	TI23	Kober 125AA	Moravia—Modřice	1.6×10^6^	GLRaV-1, GRSPaV, GVA, GVB, GRVFV, GSyV-1	GYSVd-1, HSVd
G2	TI21	Pinot noir	Bohemia—Mělník	1.5×10^6^	GPGV, GRSPaV, GFkV	GYSVd-1, HSVd
G3	TI25	Laurot	Moravia—Lednice	2.7×10^6^	GPGV, GRSPaV, GSyV-1	GYSVd-1, HSVd
G4	TI15	Müller-Thurgau	Bohemia—Karlštejn	2.1×10^6^	GLRaV-1, GRSPaV, GPGV, GRVFV, GVA	GYSVd-1, HSVd
G5	TI30	Guzal Kara	Moravia—Modřice	1.9×10^6^	GRSPaV	GYSVd-1, HSVd
G6	7/2/25	Chardonnay	Moravia—Perná	1.3×10^6^	GLRaV-1, GPGV, GRSPaV	HSVd
G7	20/5/23	Chardonnay	Moravia—Perná	8.9×10^5^	GPGV, GRSPaV, GFkV, GRVFV	GYSVd-1, HSVd
G8	22/2/23	Chardonnay	Moravia—Perná	8.2×10^5^	GPGV, GRSPaV, GRVFV	GYSVd-1, HSVd

Plant G1 is grapevine rootstock Kober 125 AA (origin *Vitis berlandieri* × *Vitis riparia*), originating from Modřice, South Moravia, coordinates 49.1252N, 16.6031E. It was tested for the first time in 2005 during the ELISA (enzyme-linked immunosorbnet assay)-based grapevine virus survey [10]. During this survey, the plant was positive in ELISA for *Grapevine leafroll-associated virus-1* (GLRaV-1), *Grapevine virus A* (GVA), *Grapevine virus B* (GVB), and *Grapevine fleck virus* (GFkV). Testing by RT-PCR also revealed infection by the *Grapevine rupestris stem pitting-associated virus* (GRSPaV) [11].

Cuttings from the plant were taken in 2005, from which a new plant was grown and cultivated at the Crop Research Institute Prague (CRI) in a screenhouse, where it was labeled as TI23. The plant did not show any symptoms of virus infection.

Plant G2, labeled as TI21 in the screenhouse of CRI (coordinates 50.0862106N, 14.2988356E), of grapevine variety Pinot Noir, had recently been bought in a market. It was selected as being representative of certified propagated material. The origin of the plant was Mělník, Central Bohemia, Czech Republic. The plant showed mild mosaic symptoms, and was positive in RT-PCR, using generic primers for tymoviruses [12].

Plant G3, labeled as TI25 in the CRI screenhouse, is a interspecific grapevine hybrid of variety Laurot, having originated from a vineyard of genetic resources held in the Mendel University in Brno, Faculty of Horticulture, Lednice, South Moravia. The cultivar is a product of crossing Merlan (Merlot × Seibel 13 666) × Fratava (Frankovka × Svatovavřinecké); while the Seibel 13666 is a complex interspecific variety, used as a donor of resistance against fungal diseases. The G3 plant is the first grapevine in the Czech Republic found to be infected by the newly emerged *Grapevine Pinot gris virus* (GPGV) [13]. The plant showed both mosaic and vein banding symptoms, which were different from those described in GPGV infection (*e*.*g*., stunting, mottling and leaf deformations) [9].

Plant G4, labeled as TI15 in the screenhouse of the CRI, is grapevine variety Müller-Thurgau, having originated in the Karlštejn Research Station of Viticulture, Central Bohemia, coordinates 49.9350611N, 14.1652414E. This Research Station is a unit of the Crop Research Institute, Prague, Czech Republic. The plant was previously found to be infected with GLRaV-1, GVA, and an unspecified tymovirus. The plant was showing symptoms of a mild mosaic, and downrolling of the leaf blades.

Plant G5, labeled as TI30 in the screenhouse of the CRI, grapevine variety Guzal Kara, having originated from Modřice, South Moravia, coordinates 49.1252N, 16.6043E, was showing symptoms of irregular and dichotomous growth, typical for nepovirus infection, although no nepovirus had thus far been detected. In 2006, the plant was also replanted into the screenhouse of the Crop Research Institute, Prague.

Plants G6-G8 were originally selected from production vineyards in Perná, South Moravia for containing different viruses based on previous commercial tests. These plants showed symptoms such as leaf mottling, mosaic, and leaf deformations; but not symptoms typical for GPGV [9]. The grapevines are currently cultivated in a screenhouse of the Crop Research Institute, Prague, coordinates 50.0862106N, 14.2988356E.

### Library preparation and sequencing

Total RNAs were extracted from 1 g of scraped phloem according to [14]. The low molecular weight RNA fraction (LMW-RNA) was isolated by polyethylenglycol precipitation [15]; small RNAs, separated by electrophoresis on polyacrylamide gel, were recovered according to [16]. Libraries of small RNAs from 8 grapevines were synthesized and sequenced with Illumina HiScanSQ (SELGE, University of Aldo Moro, Bari, Italy).

### Sequencing data analysis

The sequence quality was checked by use of fastQC-0.10.1 [17], and a FASTX-Toolkit Clipper was used for discarding of adaptors. FASTX-Toolkit Collapser was used for the distinction between unique reads and redundant ones (S1 Table). Contigs of individual reads were assembled by velvet-1.2.10 assembler [18] with a k-mer of 17. Previously, k-mer values of 15 and 17 were used for data evaluation, but k-mer 17 was selected as suitable based on more accurate blastN and blastX results. Contigs were screened for homology to known viruses by BLASTN and BLASTX against the nr database (http://www.ncbi.nlm.nih.gov/) with an e-value threshold of 10^−6^ in both. Contigs mapping to the *Vitis vinifera* L. genome, as well as those mapping to bacterial and fungal genomic fragments or their viruses were omitted from further work. A list of the potential viruses and viroids present in the analyzed samples was created, and reference sequences were selected for mapping of NGS reads. As the first choice, sequences from the RefSeq database (NCBI) were used, because they contain full-length sequences of particular viruses and viroids. For viruses with a lower homology with RefSeq-originated data, sequences with a higher homology were used for mapping, despite the fact they were not of full length. This was especially the case for GRSPaV, GVA, and GVB viruses. A list of reference sequences is presented in Table 2. Subsequently, reads were aligned with the SOAP (Short Oligonucleotide Alignment Program; [19]) package of software against the reference sequences. Mapped reads were checked by the SOAP Aligner 1.11 (BGI) allowing 2 nucleotide mismatches, and consensus sequences were obtained by CLC Genomics Workbench 6.0 (CLC Bio) software with the following parameters: Mischmatch cost = 2 (The cost of a mismatch between the read and the reference sequence); Insertion cost = 3 (The cost of an insertion in the read-causing a gap in the reference sequence); and Deletion cost = 3 (The cost of having a gap in the read). The parameters were used for Global alignment, and the reads were matched randomly. Finally, the recovered sequences of viruses and viroids were submitted to GenBank/NCBI under Acc. Nos. KP693444-KP693448 and KT000346-KT000371. Alignments and genome coverage, respectively, were visualized and estimated by Tablet 1.14 [20]. CLC Genomics Workbench 6.0 (CLC Bio) was also used for mapping of reads on reference genomes, with a mismatches tolerance of two. Comparison of SOAP Aligner 1.11 and CLC Genomics Workbench 6.0 (CLC Bio) for the mapping of reads against reference sequences was carried out, see S1 and S2 Files.

**Table 2:** List of reference sequences used for individual viruses and viroids.

Label of the vine	Virus detected	Reference sequence
**G1**	GLRaV-1	gi|366898511|ref|NC_016509.1| Grapevine leafroll-associated virus 1, complete genome
	GRSPaV	gi|9630737|ref|NC_001948.1|Grapevine rupestris stem pitting associated virus-1, complete genome
	GVA	gi|970955222|gb|KR_091864.1| Grapevine virus A, isolate CZ-Traminer, partial sequence
	GVB	gi|294336603|gb|GU733707.1| Grapevine virus B, isolate GVB-H1, complete sequence
	GRVFV	gi|57116481|gb|AY706994.1| Grapevine rupestris vein feathering virus polyprotein gene, complete cds
	GSyV-1	gi|225908238|ref|NC_012484.1| Grapevine Syrah Virus-1, complete genome
	HSVd	gi|166237160|gb|EU382210.1| Hop stunt viroid isolate CQ clone 3, complete genome
	GYSVd-1	gi|323482824|gb|HQ222363.1| Grapevine yellow speckle viroid 1, complete sequence
**G2**	GRSPaV	gi|9630737|ref|NC_001948.1|Grapevine rupestris stem pitting associated virus-1, complete genome
	GPGV	gi|339906182|ref|NC_015782.1| Grapevine Pinot gris virus, complete genome
	GFkV	gi|18138525|ref|NC_003347.1| Grapevine fleck virus, complete genome
	HSVd	gi|166237160|gb|EU382210.1| Hop stunt viroid isolate CQ clone 3, complete genome
	GYSVd-1	gi|323482824|gb|HQ222363.1| Grapevine yellow speckle viroid 1, complete sequence
**G3**	GRSPaV	gi|9630737|ref|NC_001948.1|Grapevine rupestris stem pitting associated virus-1, complete genome
	GPGV	gi|339906182|ref|NC_015782.1| Grapevine Pinot gris virus, complete genome
	GSyV-1	gi|225908238|ref|NC_012484.1| Grapevine Syrah Virus-1, complete genome
	HSVd	gi|166237160|gb|EU382210.1| Hop stunt viroid isolate CQ clone 3, complete genome
	GYSVd-1	gi|323482824|gb|HQ222363.1| Grapevine yellow speckle viroid 1, complete sequence
**G4**	GLRaV-1	gi|366898511|ref|NC_016509.1| Grapevine leafroll-associated virus 1, complete genome
	GRSPaV	gi||gb| KX274277.1|Grapevine rupestris stem pitting associated virus-1, isolate SK-30, complete genome
	GVA	gi|154431134|gb|EU_008561.1| Grapevine virus A, clone MT25/7, partial sequence
	GPGV	gi|339906182|ref|NC_015782.1| Grapevine Pinot gris virus, complete genome
	GRVFV	gi|57116481|gb|AY706994.1| Grapevine rupestris vein feathering virus polyprotein gene, complete cds
	HSVd	gi|166237160|gb|EU382210.1| Hop stunt viroid isolate CQ clone 3, complete genome
	GYSVd-1	gi|323482824|gb|HQ222363.1| Grapevine yellow speckle viroid 1, complete sequence
**G5**	GRSPaV	gi|9630737|ref|NC_001948.1|Grapevine rupestris stem pitting associated virus-1, complete genome
	HSVd	gi|166237160|gb|EU382210.1| Hop stunt viroid isolate CQ clone 3, complete genome
	GYSVd-1	gi|323482824|gb|HQ222363.1| Grapevine yellow speckle viroid 1, complete sequence
**G6**	GLRaV-1	gi|366898511|ref|NC_016509.1| Grapevine leafroll-associated virus 1, complete genome
	GRSPaV	gi|9630737|ref|NC_001948.1|Grapevine rupestris stem pitting associated virus-1, complete genome
	GPGV	gi|339906182|ref|NC_015782.1| Grapevine Pinot gris virus, complete genome
	HSVd	gi|166237160|gb|EU382210.1| Hop stunt viroid isolate CQ clone 3, complete genome
**G7**	GRSPaV	gi|9630737|ref|NC_001948.1|Grapevine rupestris stem pitting associated virus-1, complete genome
	GPGV	gi|339906182|ref|NC_015782.1| Grapevine Pinot gris virus, complete genome
	GFkV	gi|18138525|ref|NC_003347.1| Grapevine fleck virus, complete genome
	GRVFV	gi|57116481|gb|AY706994.1| Grapevine rupestris vein feathering virus polyprotein gene, complete cds
	HSVd	gi|166237160|gb|EU382210.1| Hop stunt viroid isolate CQ clone 3, complete genome
	GYSVd-1	gi|323482824|gb|HQ222363.1| Grapevine yellow speckle viroid 1, complete sequence
**G8**	GRSPaV	gi|9630737|ref|NC_001948.1|Grapevine rupestris stem pitting associated virus-1, complete genome
	GPGV	gi|339906182|ref|NC_015782.1| Grapevine Pinot gris virus, complete genome
	GRVFV	gi|57116481|gb|AY706994.1| Grapevine rupestris vein feathering virus polyprotein gene, complete cds
	HSVd	gi|166237160|gb|EU382210.1| Hop stunt viroid isolate CQ clone 3, complete genome
	GYSVd-1	gi|323482824|gb|HQ222363.1| Grapevine yellow speckle viroid 1, complete sequence

### Virus spectrum determination

After mapping the unique reads on the reference sequences, the presence of the studied viruses in grapevine vascular tissues were then determined. The data carried with BLAST and mapping analyses adjudged that viruses whose genome coverage were at least 20%, and with a sequencing depth greater than 5, were expected to be present in the sample. Further confirmation of the presence of a virus or viroid was done by RT-PCR as a second method, and a positive reaction in RT-PCR was the definitive criterium for estimating the presence of a virus or viroid.

A genome coverage threshold of 20% was used according to the authors' experience, based especially on the analysis of the presence of real viruses in examined grapevines. A sequencing depth of 5 was used according to published works [21–23].

### Confirmation of NGS results by RT-PCR

The isolation of RNA from vascular tissues was performed using a Spectrum^™^ Plant Total RNA Kit (Sigma-Aldrich, St. Louis, MO, USA). Reverse transcription was performed with a Transcriptor First Strand cDNA Synthesis Kit (Roche, Basel, Switzerland), using random hexamers for reaction priming according to the manufacturer's suggestions. FastStart Taq DNA Polymerase (Roche, Basel, Switzerland) was used for PCR. The conditions and primers used for the detection of individual viruses and viroids are listed in Table 3. Primers for the detection of GRVFV and HSVd were designed in this study. They were primarily based on the NGS sequences created from this study, with regard to the sequences available in GenBank for the respective virus or viroid; as well as a view toward the use of the primers for future routine diagnostics. Primer3Plus [24] was used for the primer design with the default parameters.

**Table 3:** Description of the primers and PCR conditions used for the detection of grapevine viruses.

Virus	Primers	Expected product size (bp)	PCR cycling conditions	Reference
GLRaV-1	5′ TGGCATCGTTGCTAAATTGAG 3′ ^A^	175	95°C/15 min, 40× (94°C/30 s, 53°C/30 s, 72°C/1 min), 72°C/10 min	[11]
	5′ AATCCTATGCGTCAGTATGC 3′ ^S^			
GRSPaV	5′ CACATRTCATCVCCYGCAAA 3′ ^A^	476	95°C/15 min, 40× (94°C/55 s, 50°C/55 s, 72°C/55 s), 72°C/10 min	[25]
	5′ AGRYTTAGRGTRGCTAARGC 3′ ^S^			
GPGV	5′ CTGAGAAGCATTGTCCCATC 3′ ^A^	303	95°C/15 min, 40× (94°C/55 s, 55°C/55 s, 72°C/55 s), 72°C/10 min	[13]
	5′ ATTGCGGAGTTGCCTTCAAG 3′ ^S^			
GVA	5′ TCGAACATAACCTGTGGCTC 3′ ^A^	271	95°C/15 min, 40× (94°C/45 s, 50°C/45 s, 72°C/1 min), 72°C/10 min	[26]
	5′ GAGGTAGATATAGTAGGACCT 3′ ^S^			
GVB	5′ GTGCTAAGAACGTCTTCACAGC 3′ ^A^	158	95°C/15 min, 40× (94°C/45 s, 50°C/45 s, 72°C/1 min), 72°C/10 min	[27]
	5′ AGTAGCCCTTCGTTTAGCCGC 3′ ^S^			
GFkV	5′ CGACGCAGGCGTCATTGCG 3′ ^A^	520	95°C/15 min, 40× (94°C/55 s, 55°C/55 s, 72°C/55 s), 72°C/10 min	[28]
	5′ CCGTCTGCTGACCAGCCTG 3′ ^S^			
GSyV-1	5′ CGACGCAGGCGTCATTGCG 3′ ^A^	374	95°C/15 min, 40× (94°C/40 s, 56°C/40 s, 72°C/50 s), 72°C/10 min	[29]
	5′ CCGTCTGCTGACCAGCCTG 3′ ^S^			
GRVFV	5′ ATGTTGGTCTTCACGTGSAC 3′ ^A^	266	95°C/15 min, 40× (94°C/40 s, 55°C/40 s, 72°C/50 s), 72°C/10 min	this study
	5′ GTCCTCGGCCCAGTGAAAA 3′ ^S^			
HSVd	5′ GTTGGAAGACGAACCGAGAG 3′ ^A^	171	95°C/15 min, 40× (94°C/40 s, 55°C/40 s, 72°C/50 s), 72°C/10 min	this study
	5′ GGGCAACTCTTCTCAGAATCC 3′ ^S^			
GYSVd-1	5′ ACTTCATGGTGGTGCCGGTG 3′ ^A^	374	95°C/15 min, 40× (94°C/55 s, 55°C/55 s, 72°C/55 s), 72°C/10 min	[30]
	5′ CCAATGGGTCGCACTTGTTG 3′ ^S^			

Primers: ^A^ antisense ^S^ sense

In the case of unclear results, and a first record of the pathogens, the products of RT-PCR, were cloned into pGEM-T Easy vector and sequenced. At least five clones from every sample were commercially sequenced. The resulting sequences were compared, the genetic divergence was estimated, and a phylogenetic tree was constructed using the MEGA7 program [31] to ensure a proper determination of the amplified genomic fragment.

## Results

### Viruses and viroids detection by NGS

Plants of various varieties and geographical origins were included in the analyzed set; primarily from Moravia (localities Modřice, Lednice, and Perná), but also from wine regions in Bohemia (localities Karlštejn and Mělník). Libraries representative of the sRNA populations extracted from grapevines G1-G8, and sequenced by Illumina technology, contained G1 = 1.6×10^6^, G2 = 1.5×10^6^, G3 = 2.7×10^6^, G4 = 2.1×10^6^, G5 = 1.9×10^6^, G6 = 1.3×10^6^, G7 = 8.9×10^5^, G8 = 8.2×10^5^ unique reads, respectively (Table 1). Further data (e.g., numbers of reads after clipping by fastQC and numbers of unique reads) are available as S1 Table. *De novo* assembly of sequenced reads, and a BLAST search for homologies of the obtained contigs led to the identification of 8 different viruses and 2 viroids in a set of the 8 grapevines (Table 1). The eight detected viruses belong to several families: *Tymoviridae* (*Grapevine fleck virus*, *Grapevine rupestris vein feathering virus*, *Grapevine Syrah virus 1*), *Closteroviridae* (*Grapevine leafroll-associated virus 1*), and *Betaflexiviridae* (*Grapevine Pinot gris virus*, *Grapevine rupestris stem pitting-associated virus*, *Grapevine virus A*, *Grapevine virus B*); whereas, the two viroids (*Hop stunt viroid* and *Grapevine yellow speckle viroid 1*) belong to the family *Pospiviroidae*.

The sequences obtained were submitted to GenBank NCBI under the following accession numbers: KT000353-KT000360 (HSVd), KP693444-KP693448 (GPGV), KT000346-KT000352 (GYSVd-1), KT000361-KT000362 (GFkV), KT000363-KT000365 (GLRaV-1), and KT000366-KT000371 (GRSPaV). Sequences of GVA, GVB, GRVFV, and GSyV-1 were not completed because of the low coverage of the full-length reference sequences.

The results of sRNA sequencing were checked by RT-PCR detection; thus meaning that all detected pathogens (viruses and viroids) had their presence proven in the tested materials by at least two methods (see discussion and Table 4). All mapped reads were normalized to 1 million reads in order to enable a comparative analysis of a single virus' presence in the vascular tissues (S1 and S2 Files, S1 Table).

**Table 4:** Number of contigs, genome coverage, and RT-PCR detections. There are listed total assembled mapped reads that are not normalized to 1 million reads.

Label of the vine	Virus detected	contigs identified by BLAST	reads assembled by SOAP	coverage (reads mapped to reference) %	confirmation by RT-PCR
**G1**	GLRaV-1	172	38 984	74	positive
	GRSPaV	113	3 208	46	positive
	GVA	71	3 052	99	positive
	GVB	83	16 856	99	positive
	GRVFV	89	4 961	33	positive
	GSyV-1	2	1 086	68	positive
	HSVd	5	1 132	90	positive
	GYSVd-1	1	805	91	positive
**G2**	GRSPaV	169	3 776	97	positive
	GPGV	72	9 285	98	positive
	GFkV	123	14 399	90	positive
	HSVd	4	1 423	94	positive
	GYSVd-1	4	3 273	100	positive
**G3**	GRSPaV	185	7 755	100	positive
	GPGV	81	14 812	98	positive
	GSyV-1	37	1 292	24	positive
	HSV-1	6	1 313	100	positive
	GYSVd-1	3	2 483	100	positive
**G4**	GLRaV-1	153	35 016	78	positive
	GRSPaV	71	1 222	25	positive
	GVA	58	5 900	100	positive
	GPGV	2	238	18	positive
	GRVFV	120	4 580	29	positive
	HSVd	6	1 297	100	positive
	GYSVd-1	6	2 800	100	positive
**G5**	GRSPaV	68	10 237	100	positive
	HSV	199	1 395	90	positive
	GYSVd-1	0	95	100	positive
**G6**	GLRaV-1	162	30 117	74	positive
	GRSPaV	3	6 111	99	positive
	GPGV	19	4 003	99	positive
	HSV	7	1 355	100	positive
**G7**	GRSPaV	35	4 288	94	positive
	GPGV	39	5 942	95	positive
	GFkV	60	30 408	97	positive
	GRVFV	23	9 376	22	positive
	HSVd	9	1 384	100	positive
	GYSV-1	8	2 331	100	positive
**G8**	GRSPaV	18	2 935	97	positive
	GPGV	24	4 918	98	positive
	GRVFV	54	9 560	29	positive
	HSVd	6	1 025	100	positive
	GYSVd-1	9	2 199	100	positive

### Comparison of read mapping efficiency using SOAP-1.11 aligner and CLC Genomics Workbench 6.0

To compare the read mapping efficiency of two frequently used programs (SOAP-1.11 [19] aligner, and CLC Genomics Workbench 6.0 (CLC Bio)), the obtained reads were mapped on reference sequences using both methods. The number of reads was mapped on reference sequences and referenced to unique reads per 1 million. After the blast contigs analysis, a database of 18 viruses potentially present in the tested vascular tissues was created. In the case of unique reads, mapping to 13 reference sequences, 244,859 reads were mapped in SOAP (S1 File); this being a significantly higher number than when using CLC Genomics Workbench, with 144,899 reads (S2 File). Based on these data, the SOAP aligner seems to represent the more sensitive solution for this type of analysis compared to the CLC mapping algorithm.

### Description of detected viruses

#### Grapevine rupestris stem pitting—associated virus

*Grapevine rupestris stem pitting—associated virus* (GRSPaV), belonging to the genus *Foveavirus*, family *Betaflexiviridae*, was confirmed to be present in all eight plants. A high coverage of mapped reads occurred in the majority of vines (6 out of 8) ranging from 97% to 100%; with low coverage in plants G1 and G4, reaching only 46% and 25%, respectively. The greatest amount, corresponding to sRNA GRSPaV, was detected in the G3 (Laurot, locality Lednice/Moravia) and G5 plants (Guzal Kara, locality Modřice/Moravia), with 100% coverage of the genome. Regarding the high level of GRSPaV genome variability, we used as a reference sequence KX274277.1 for mapping on sample G4 with 25% resulting genome coverage; the mapping on reference NC_001948.1 provided genome coverage that was too weak (only 16%); however, the other results suggested the presence of GRSPaV.

#### Grapevine Pinot gris virus

*Grapevine Pinot gris virus* (GPGV), genus *Trichovirus*, family *Betaflexiviridae*, is a newly-emerged virus, recently identified in Italy [3], and subsequently in several other countries [9, 32, 33, 34, 35], as well as in the Czech Republic [13, 36]. GPGV was found to be present in a high number of the tested plants. It was reliably detected in 5 out of 8 vines, and with a genome coverage ranging from 95% (G7) to 98% (G8). Plant G4 was an exception with lower coverage (18%). RT-PCR even confirmed the presence of GPGV in this plant. Considering the very small number of plants analyzed within this work, the finding of 5 positive plants suggests the likely widespread occurrence of GPGV in Czech vineyards, recently confirmed by [36]. The sudden emergence of GPGV in a number of different countries suggests a longer presence of the virus in those regions where it had been unnoticed prior.

#### Grapevine virus A

*Grapevine virus A* (GVA), genus *Vitivirus*, family *Betaflexiviridae*, was detected in plants G1 and G4. GVA is a common grapevine virus in the Czech Republic [10]. It had already been detected in both the G1 and G4 plants before the NGS analysis, and was confirmed by RT-PCR during the present experiments. Genomic coverage of GVA, using a partial sequence from Central Europe, reached 99–100%. However, using reference sequences, the coverage was as low as 23–25%; showing the need to use reference sequences phylogenetically close to the analyzed ones.

#### Grapevine virus B

*Grapevine virus B* (GVB), genus *Vitivirus*, family *Betaflexiviridae*, was detected in plant G1. The virus is very rare in the Czech Republic [10]. In fact, this is the only plant infected with GVB ever found in the Czech Republic through all of the previous surveys.

#### Grapevine leafroll associated virus 1

*Grapevine leafroll associated virus 1* (GLRaV-1), genus *Ampelovirus*, family *Closteroviridae*, was detected in plants G1, G4, and G6. The virus had the greatest number of reads among all of the viruses. This is caused by the fact that it has the largest genome (more than 18 kb) compared to *e*.*g*., the 6–8 kb in fovea-, tricho-, and tymoviruses. GLRaV-1 occurrence is common in the Czech Republic [10].

#### Grapevine fleck virus

*Grapevine fleck virus* GFkV, genus *Maculavirus*, family *Tymoviridae*, was detected in plants G2 and G7. GFkV is a common grapevine virus in the Czech Republic according to the previous surveys [10].

#### Grapevine rupestris vein feathering virus

*Grapevine rupestris vein feathering virus* (GRVFV), genus *Marafivirus*, family *Tymoviridae*, was detected in plants G1, G4, G7, and G8. RT-PCR with primers designed on the contigs and sequencing of the cloned PCR product confirmed the presence and proper taxonomic identity of the virus in all of the plants. The sequence of the PCR product was submitted into GenBank under reference number KX465108.

This is the first confirmation of the presence of GRVFV in the Czech Republic.

#### Grapevine Syrah virus 1

*Grapevine Syrah virus 1* (GSyV-1), genus *Marafivirus*, family *Tymoviridae*, was detected in plants G1 and G3. The presence of this virus on the examined plants was also confirmed by [29].

#### Grapevine yellow speckle viroid 1

*Grapevine yellow speckle viroid 1* (GYSVd-1), genus *Apscaviroid*, family *Pospiviroidae*, was found to be present in 7 out of the 8 plants. To our knowledge, this is the first described occurrence of *Grapevine yellow speckle viroid 1* in the Czech Republic. The viroid is reported to occur worldwide; *e*.*g*., in Italy [9], USA [37], India [38], and New Zealand [30].

#### Hop stunt viroid

The next viroid, *Hop stunt viroid* (HSVd), genus *Hostuviroid*, family *Pospiviroidae*, was detected in all eight grapevine plants. HSVd was earlier detected in Czech grapevines by [39]. However, the samples tested in the study mentioned were taken in the vicinity of hops gardens in northern Bohemia and the Znojmo wine-producing region. Our results showed that the viroid is also present in plants grown in other Czech wine-producing regions, suggesting that the proximity of hops is not necessary for this pathogen to be present in grapevines.

### Symptoms of viral disease on examined grapevines and their relationship to detected pathogens

Plant G1, which is grapevine rootstock Kober 125AA, does not show symptoms of virus disease, although it is infected with at least 7 viruses and viroids. As this genotype is an indicator for rugose wood complex viral disease, it should show some symptoms, as the plant is infected with several viruses responsible for expression of the disease mentioned. However, after grafting inoculum from this plant into the sensitive LN33 indicator, symptoms will appear [11]. It is too speculative to estimate the relationships of the 7 detected viruses and viroids on the appearance or disappearance of the final symptoms on individual genotypes of *Vitis* sp.

Plant G2, showing symptoms of a mild mosaic, was found to be infected with several pathogens including GPGV (Table 1). Mosaic is not a typical symptom of GPGV as described by [9], but also not of the other viruses and viroids present. Since no typical symptoms of GPGV have so far been found in the Czech Republic (although it seems the virus is present), we should expect milder symptoms for GPGV such as the mosaic in plant G2.

Plant G3, showing symptoms of mosaic and vein banding, was known to contain GPGV. The presence of GPGV was confirmed together with other viruses and viroids. The typical symptoms of GPGV were not observed.

Plant G4, showing symptoms of downrolling of its leaf blades, was found to contain GLRaV-1, GVA, GRSPaV, and viroids. GLRaV-1 is the cause of the downrolling of the leaf blades.

Plant G5, showing dichotomous growth, was expected to contain some nepovirus. However, no nepovirus was detected by NGS, only GRSPaV and viroids. The symptoms are probably due to another endogenous factor causing a phytohormone imbalance [40].

Plants G6 to G8 were showing symptoms of leaf mottling and deformations, but not those typical for GPGV. All three had a confirmed infection by NGS of GPGV. Since all three come from the cultivar Chardonnay, we can conclude that GPGV can cause symptoms of leaf mottling and deformations in this cultivar under Czech conditions.

### Confirmation of virus and viroid presence using RT-PCR

Based on the results of sRNA NGS detection, RT-PCR with specific primers to the corresponding pathogens were applied to RNA isolated from vascular tissues to confirm virus and viroid presence in the analyzed plants. The results are given in Table 4.

Using the RT-PCR method, GYSVd-1 in plant G6 was not detected, while the mapping of reads covered 57% of the reference sequence. Additionally, not one contig was identified to be of GYSVd-1 origin. The other seven plants were positive for the viroid in RT-PCR, with coverage ranging from 91–100%. We can conclude that the viroid is not present in plant G6.

GPGV was detected by RT-PCR, even in plant G4; although the coverage was only 18%, which is under the 20% threshold. The numbers of mapped reads are shown in S1 and S2 Files.

GFkV was detected by NGS in plants G2 and G7, and also confirmed by RT-PCR. In its first occurrence in the Czech Republic, another virus from the family *Tymoviridae*, *Grapevine rupestris vein feathering virus* (GRVFV) was detected in plant G1. Its presence was confirmed by RT-PCR and sequencing of the PCR product. As mentioned in the introduction about plants tested in the present work, the G1 plant was originally positive in ELISA for GFkV. Since no GFKV is present in plant G1 (but GRVFV is), the antibodies were also probably accidentally raised against another virus other than GFkV. GRVFV was also detected in plants G4, G7, and G8.

For all of the reasons delineated, and due to the end-point nature of RT-PCR, as well as the massively parallel nature of sRNA NGS, sensitivity comparisons of the two methods can be very difficult. This subject should continue to be studied diligently. Further, it needs to be ascertained what kind of coverage and sequencing depth is sufficient to determine a positive sample. A comprehensive database of virus sequences should be used for NGS contig identification, and the one with the best matching score should then be used for genome mapping. For many grapevine viruses, full-length genome sequences from the Central European region are still missing.

## Discussion and Conclusion

The diagnostics of RNA pathogens (viruses and viroids) was carried out using sRNA sequencing. This method seems very promising, using the metagenomic approach [3–4], considering the possibility of simultaneous detection of all of the RNAi influenced pathogens present.

The plants used in this study were all tested and previously found positive for the presence of some RNA viruses. However, the spectrum range of RNA pathogens detected was still unexpected. In every one from the evaluated set of 8 plants, at least 3 RNA pathogens were detected. This study also provides an unique comparison of various varieties, grown in different wine regions of the Czech Republic. The relatively high occurrence of GLRaV-1 and GFkV in the Czech Republic is a well-known fact [28, 41]. However, this was not the case for GPGV, because of the recent emergence of the virus. Recently, a new study has been published [29], showing the wide spread of this virus within the Czech Republic.

Distribution of foveavirus GRSPaV in the Czech Republic has not yet been studied; however, thus far, our experience with grapevine virus detection shows that its occurrence in Czech grapevines will be very high [11, 42].

This study is also the first work to prepare libraries for sRNA sequencing from grapevine vascular tissues. [43] used wood tissue to prepare a sRNA library. Their work was not focused on the detection of plant pathogens, but rather on the research of the gene regulatory network, in order to provide profound biological insights into the regulation of xylem development. Using vascular tissues directly as the source of RNA material is much more advantageous due to the more balanced levels of RNA concentrations within the tissues [44–45]. Some of the viral pathogens also reproduce in vascular tissues; this is the case for GLRaV-1 included in the present work. Also, unlike the leaf tissue based detection, samples of vascular tissues can be taken throughout the year. The sRNA detection sensitivity is also very high [4]; probably the highest of the available detection methods.

The virus detection percentage rates obtained in this study among the set of 8 plants from 5 localities are: GRSPaV—100%, GPGV—75%, GRVFV—50%, GLRaV-1–38%, GFkV—25%, GVA—25%, GSyV-1–25%, and GVB—13%. Viroid HSVd was detected in 100% of the plants, GYSVd-1 in 88% of the plants. This result is especially interesting due to the different geographical origins of the plants tested. According to the requirements of Czech legislation (Act No. 219/2003 Coll., Ministerial Decree No. 332/2006 Coll.), plant propagation materials have to be tested for GFLV, ArMV, GLRaV-1, and GLRaV-3 (as well as for GFkV in the case of rootstock mother plants), which are all widespread in the Czech Republic [10]. However, this study shows lower percentage rates of these viruses' presence in vascular tissue compared to GRSPaV, GPGV, and GRVFV; as well as for the viroids GYSVd-1 and HSVd, which may or may not be caused by the limited number of tested samples. Lower percentage of controlled pathogens could be also the effect of the fact they are controlled. As already addressed in the previous work [10], grapevine clones affected with really detrimental viruses were removed during many years of negative selections done on research stations maintaining prebasic propagation material of all Czech grapevine clones. The relatively high expense needed to test the plants by NGS is currently a limiting factor for the wider use of this method.

Use of the coverage value set at 20% proved to be a good threshold for estimating virus presence, taking into consideration use of suitable reference sequences for mapping, mostly the one giving the best identity during the BLAST search.

The fact that all of the tested plants turned out to be positive for the presence of viroids is another interesting observation of this study.

As a general conclusion, the results presented in this study demonstrate the great importance of correct and proper processing of NGS data, as well as the need for localized reference data on viruses and viroids.

## Supporting Information

S1 File Numbers of reads mapped on reference viral sequences—SOAP. (PPTX)

S2 File Numbers of reads mapped on reference viral sequences—CLC. (PPTX)

S1 Table FastQC results. (PPTX)
