# Supplementary material for: Comprehensive Virus Detection Using Next Generation Sequencing in Grapevine Vascular Tissues of Plants Obtained from the Wine Regions of Bohemia and Moravia (Czech Republic)
Source: PLoS One. 2016 Dec 13;11(12):e0167966. doi: 10.1371/journal.pone.0167966 (PMC5154529; doi:10.1371/journal.pone.0167966)
Supplement: S1 File — (PPTX) [file pone.0167966.s001.pptx]

## Slide 1
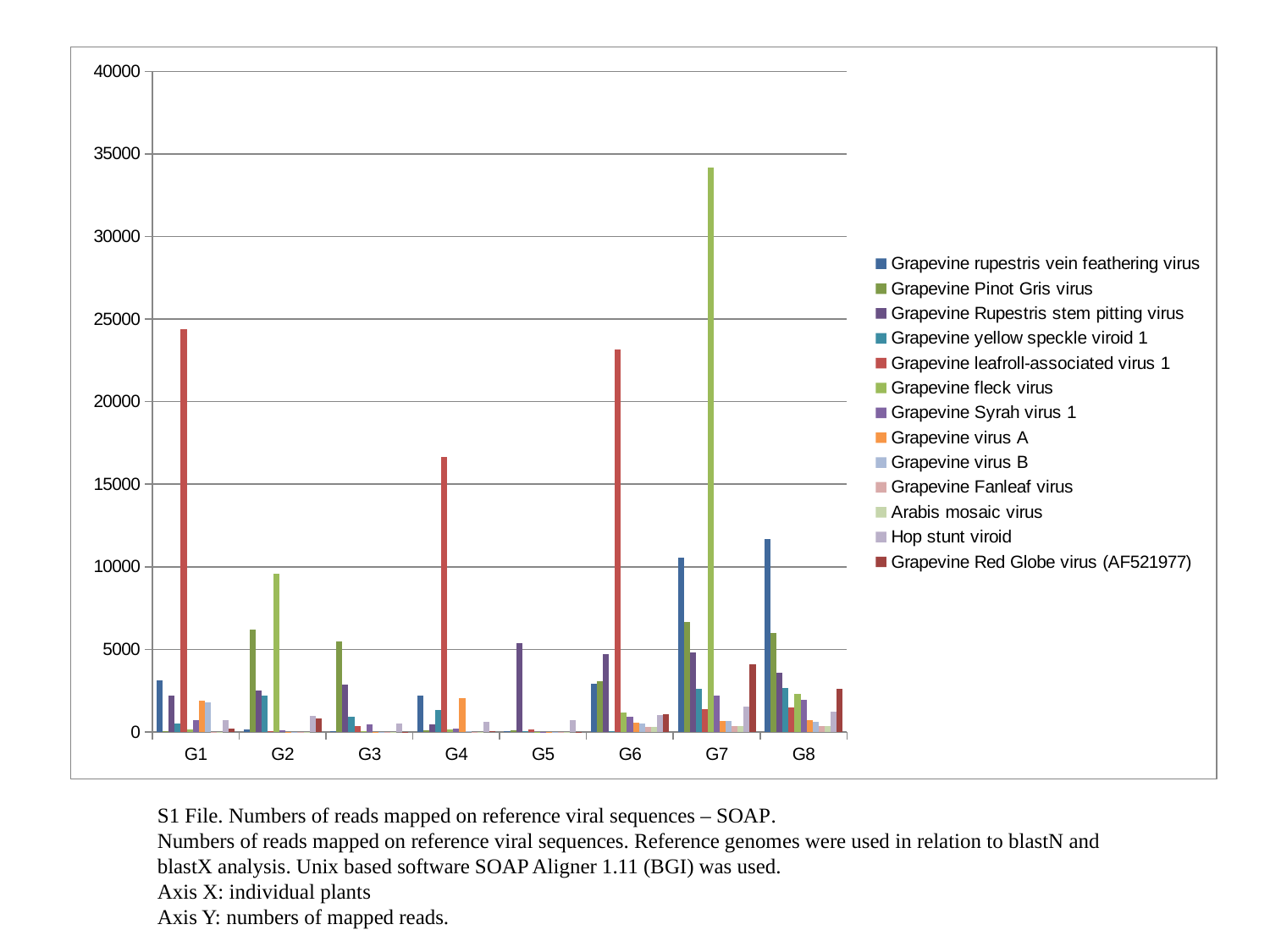

### Chart
| Category | Grapevine rupestris vein feathering virus | Grapevine Pinot Gris virus | Grapevine Rupestris stem pitting virus | Grapevine yellow speckle viroid 1 | Grapevine leafroll-associated virus 1 | Grapevine fleck virus | Grapevine Syrah virus 1 | Grapevine virus A | Grapevine virus B | Grapevine Fanleaf virus | Arabis mosaic virus | Hop stunt viroid | Grapevine Red Globe virus (AF521977) |
|---|---|---|---|---|---|---|---|---|---|---|---|---|---|
| G1 | 3112.5 | 65.0 | 2205.0 | 503.0 | 24365.0 | 159.375 | 734.375 | 1907.5 | 1766.25 | 4.0 | 2.0 | 708.0 | 225.0 |
| G2 | 148.0 | 6190.0 | 2517.33333333333 | 2182.0 | 72.0 | 9599.33333333333 | 118.666666666667 | 11.3333333333333 | 14.0 | 3.33333333333333 | 2.0 | 948.666666666667 | 831.333333333333 |
| G3 | 43.3333333333333 | 5485.92592592593 | 2872.22222222222 | 919.62962962963 | 364.444444444444 | 38.5185185185185 | 478.518518518519 | 12.962962962963 | 7.40740740740741 | 3.7037037037037 | 1.48148148148148 | 486.296296296296 | 5.18518518518519 |
| G4 | 2180.95238095238 | 113.333333333333 | 446.666666666667 | 1333.33333333333 | 16674.2857142857 | 128.571428571429 | 209.047619047619 | 2024.7619047619 | 25.2380952380952 | 5.23809523809524 | 2.38095238095238 | 617.619047619048 | 58.5714285714286 |
| G5 | 52.6315789473684 | 78.421052631579 | 5387.89473684211 | 50.0 | 151.052631578947 | 23.6842105263158 | 12.1052631578947 | 20.5263157894737 | 4.21052631578947 | 3.15789473684211 | 3.15789473684211 | 734.21052631579 | 3.15789473684211 |
| G6 | 2901.53846153846 | 3079.23076923077 | 4700.76923076923 | 59.2307692307692 | 23166.9230769231 | 1191.53846153846 | 928.461538461539 | 553.076923076923 | 533.076923076923 | 322.307692307692 | 280.769230769231 | 1042.30769230769 | 1051.53846153846 |
| G7 | 10534.8314606742 | 6676.40449438202 | 4817.97752808989 | 2619.10112359551 | 1397.75280898876 | 34166.2921348315 | 2212.3595505618 | 687.640449438202 | 659.550561797753 | 349.438202247191 | 375.280898876404 | 1555.05617977528 | 4114.60674157303 |
| G8 | 11658.5365853659 | 5997.56097560976 | 3579.26829268293 | 2681.70731707317 | 1459.75609756098 | 2286.58536585366 | 1940.24390243902 | 690.243902439024 | 601.219512195122 | 345.121951219512 | 341.463414634146 | 1250.0 | 2591.46341463415 |S1 File. Numbers of reads mapped on reference viral sequences – SOAP.
Numbers of reads mapped on reference viral sequences. Reference genomes were used in relation to blastN and blastX analysis. Unix based software SOAP Aligner 1.11 (BGI) was used.
Axis X: individual plants
Axis Y: numbers of mapped reads.

## Slide 2
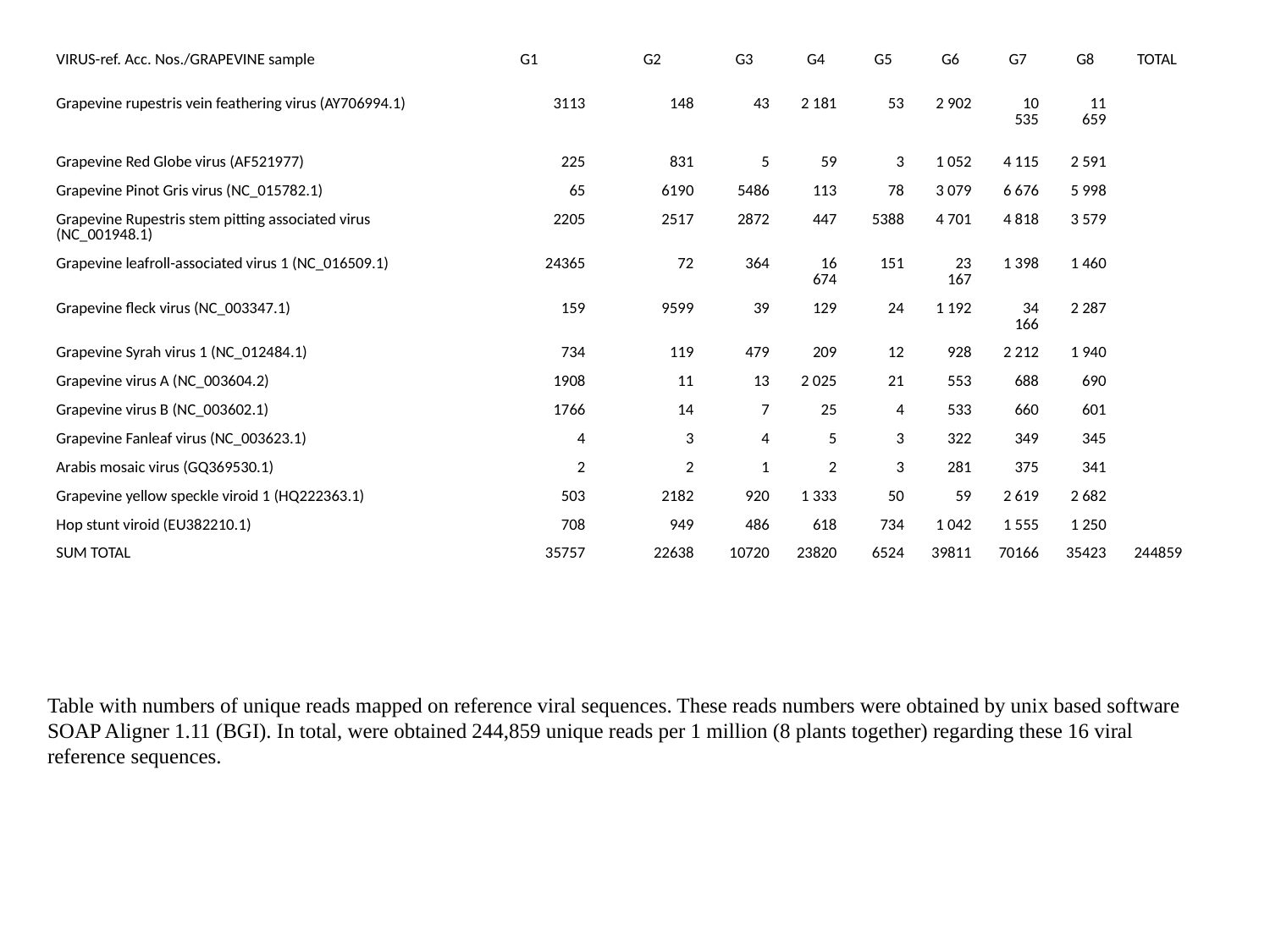

| VIRUS-ref. Acc. Nos./GRAPEVINE sample | G1 | G2 | G3 | G4 | G5 | G6 | G7 | G8 | TOTAL |
| --- | --- | --- | --- | --- | --- | --- | --- | --- | --- |
| Grapevine rupestris vein feathering virus (AY706994.1) | 3113 | 148 | 43 | 2 181 | 53 | 2 902 | 10 535 | 11 659 | |
| Grapevine Red Globe virus (AF521977) | 225 | 831 | 5 | 59 | 3 | 1 052 | 4 115 | 2 591 | |
| Grapevine Pinot Gris virus (NC\_015782.1) | 65 | 6190 | 5486 | 113 | 78 | 3 079 | 6 676 | 5 998 | |
| Grapevine Rupestris stem pitting associated virus (NC\_001948.1) | 2205 | 2517 | 2872 | 447 | 5388 | 4 701 | 4 818 | 3 579 | |
| Grapevine leafroll-associated virus 1 (NC\_016509.1) | 24365 | 72 | 364 | 16 674 | 151 | 23 167 | 1 398 | 1 460 | |
| Grapevine fleck virus (NC\_003347.1) | 159 | 9599 | 39 | 129 | 24 | 1 192 | 34 166 | 2 287 | |
| Grapevine Syrah virus 1 (NC\_012484.1) | 734 | 119 | 479 | 209 | 12 | 928 | 2 212 | 1 940 | |
| Grapevine virus A (NC\_003604.2) | 1908 | 11 | 13 | 2 025 | 21 | 553 | 688 | 690 | |
| Grapevine virus B (NC\_003602.1) | 1766 | 14 | 7 | 25 | 4 | 533 | 660 | 601 | |
| Grapevine Fanleaf virus (NC\_003623.1) | 4 | 3 | 4 | 5 | 3 | 322 | 349 | 345 | |
| Arabis mosaic virus (GQ369530.1) | 2 | 2 | 1 | 2 | 3 | 281 | 375 | 341 | |
| Grapevine yellow speckle viroid 1 (HQ222363.1) | 503 | 2182 | 920 | 1 333 | 50 | 59 | 2 619 | 2 682 | |
| Hop stunt viroid (EU382210.1) | 708 | 949 | 486 | 618 | 734 | 1 042 | 1 555 | 1 250 | |
| SUM TOTAL | 35757 | 22638 | 10720 | 23820 | 6524 | 39811 | 70166 | 35423 | 244859 |
Table with numbers of unique reads mapped on reference viral sequences. These reads numbers were obtained by unix based software SOAP Aligner 1.11 (BGI). In total, were obtained 244,859 unique reads per 1 million (8 plants together) regarding these 16 viral reference sequences.
