# Supplementary material for: Comprehensive Virus Detection Using Next Generation Sequencing in Grapevine Vascular Tissues of Plants Obtained from the Wine Regions of Bohemia and Moravia (Czech Republic)
Source: PLoS One. 2016 Dec 13;11(12):e0167966. doi: 10.1371/journal.pone.0167966 (PMC5154529; doi:10.1371/journal.pone.0167966)
Supplement: S1 Table — (PPTX) [file pone.0167966.s003.pptx]

## Slide 1
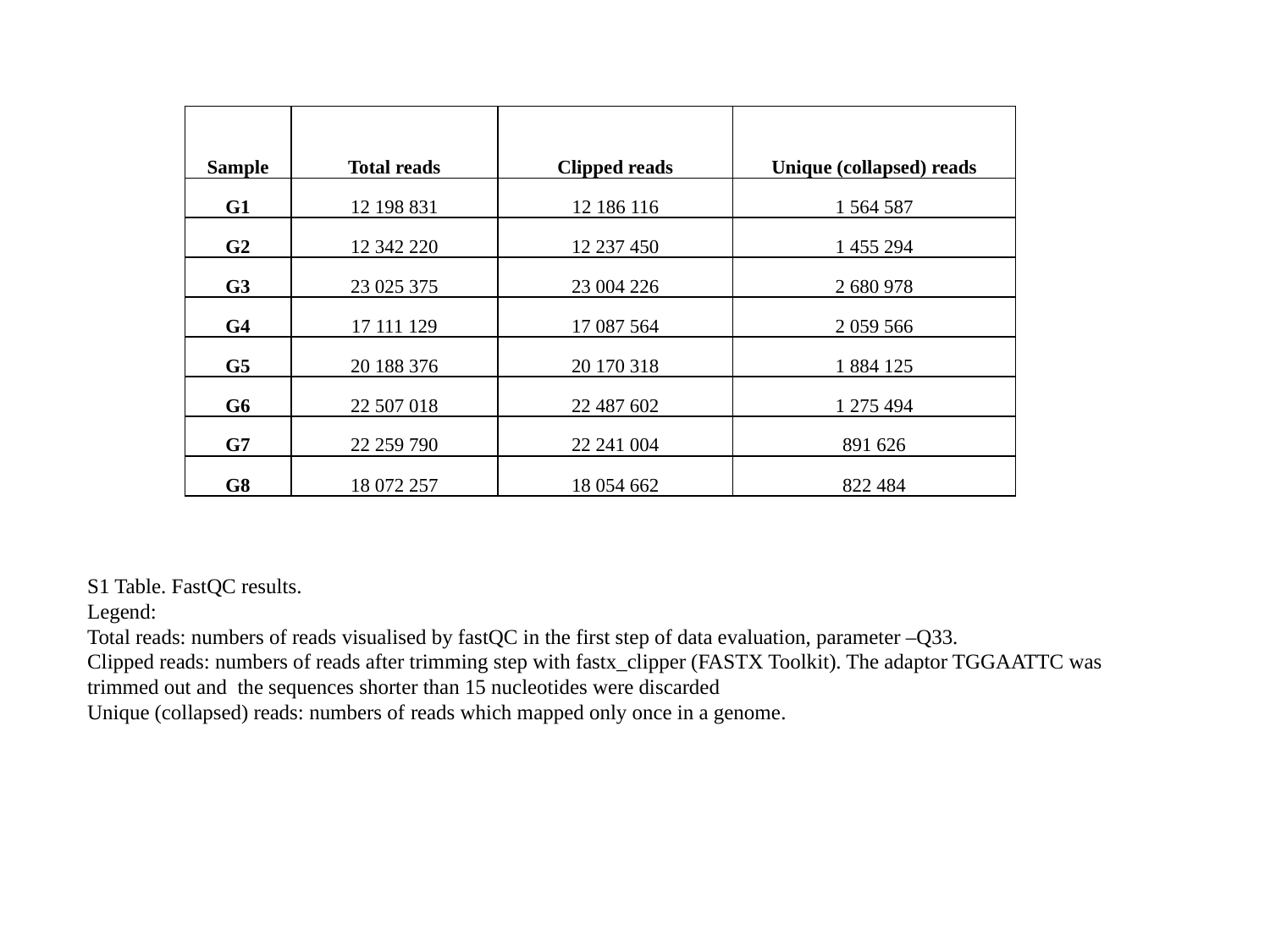

| Sample | Total reads | Clipped reads | Unique (collapsed) reads |
| --- | --- | --- | --- |
| G1 | 12 198 831 | 12 186 116 | 1 564 587 |
| G2 | 12 342 220 | 12 237 450 | 1 455 294 |
| G3 | 23 025 375 | 23 004 226 | 2 680 978 |
| G4 | 17 111 129 | 17 087 564 | 2 059 566 |
| G5 | 20 188 376 | 20 170 318 | 1 884 125 |
| G6 | 22 507 018 | 22 487 602 | 1 275 494 |
| G7 | 22 259 790 | 22 241 004 | 891 626 |
| G8 | 18 072 257 | 18 054 662 | 822 484 |
S1 Table. FastQC results.
Legend:
Total reads: numbers of reads visualised by fastQC in the first step of data evaluation, parameter –Q33.
Clipped reads: numbers of reads after trimming step with fastx_clipper (FASTX Toolkit). The adaptor TGGAATTC was trimmed out and the sequences shorter than 15 nucleotides were discarded
Unique (collapsed) reads: numbers of reads which mapped only once in a genome.
